# Identification of Degradation Products and a Stability-Indicating RP-HPLC Method for the Determination of Flupirtine Maleate in Pharmaceutical Dosage Forms

**DOI:** 10.3797/scipharm.1310-01

**Published:** 2013-12-09

**Authors:** Ramalingam Peraman, K. V. Lalitha, Naga Mallikarjuna Raja, Hari Babu Routhu

**Affiliations:** 1 Division of Pharmaceutical Analysis and Quality Assurance, Centre for Pharmaceutical Research (CPR), Raghavendra Institute of Pharmaceutical Education and Research, Anantapuramu, Andhra Pradesh, 515721, India.; 2 Aurigene Discovery Technologies Limited, Hyderabad, 500049, India.

**Keywords:** Flupirtine maleate, Stability, RP-HPLC, Characterization, Spectral Studies

## Abstract

In this stability-indicating, reversed-phase high-performance liquid chromatographic method for flupiritine maleate, forced degradation has been employed and the formed degradants were separated on a C_18_ column with a 80:20% v/v mixture of methanol-water containing 0.2% (v/v) triethylamine; the pH was adjusted to 3.1. The flow rate was 1 mLmin^−1^ and the photodiode array detection wavelength was 254 nm. Forced degradation of the drug was carried out under acidic, basic, thermal, photolytic, peroxide, and neutral conditions. Chromatographic peak purity data indicated no co-eluting peaks with the main peaks. This method resulted in the detection of seven degradation products (D1–D7). Among these, three major degradation products from acidic and basic hydrolysis were identified and characterized by ^1^H-NMR, ^13^C-NMR, and mass spectral data. The method was validated as per International Conference on Harmonization guidelines (Q2). The linearity of the method was in the concentration range of 20–120 μgmL^−1^. The relative standard deviations for intra- and interday precision were below 1.5%. The specificity of the method is suitable for the stability-indicating assay.

## Introduction

**Fig. 1:**
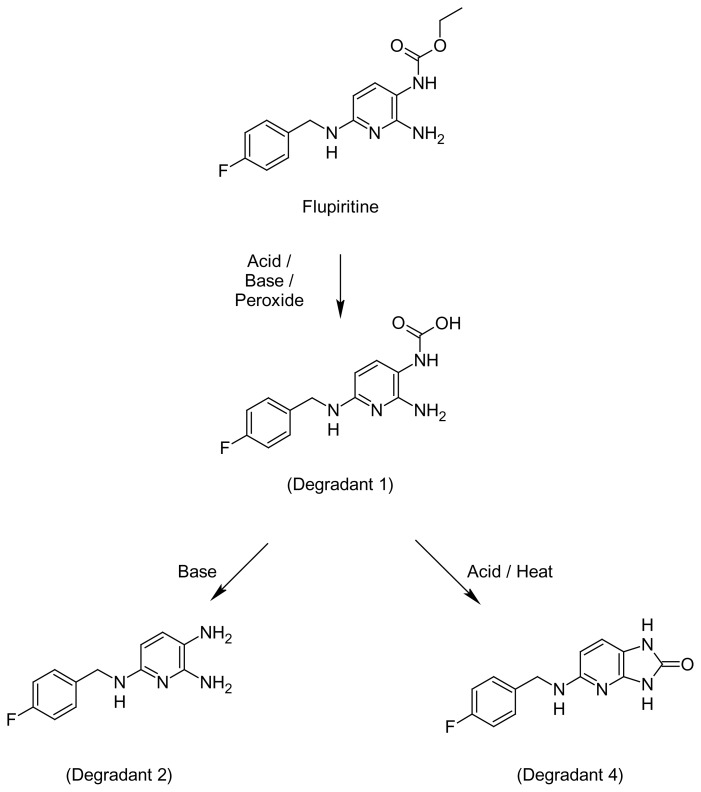
Structure of flupiritine and its identified impurities D1, D2, and D4

Flupirtine maleate, ethyl {2-amino-6-[(4-fluorobenzyl)amino]pyridin-3-yl}carbamate (FLU, Fig. 1), is a selective neuronal potassium channel opener and neuromuscular dopamine adenosine (NMDA) receptor antagonist. It is used as a non-opioid analgesic in the clinical management of acute and chronic pain. Flupirtine has approximately one-third of the analgesic potency of codeine and possesses muscle relaxant properties [1]. Chemically, FLU is a carbamate, possessing both ester and amide linkages in the chemical structure, which are highly susceptible to hydrolytic degradation under a variety of stress environments. The current practices in stability studies, forced degradation studies, and the characterization of major degradation products, are playing an important role in the development of the stability-indicating assay method (SIAM). Results of the forced degradation studies facilitate the development of SIAM, design of formulation, choice of storage conditions, and the understanding of the intrinsic stability of the drug molecule [2].

Literature revealed that few bio-analytical methods such as LC-MS/MS [3–5], HPLC [6, 7], and assay methods for the quantification of FLU in capsules using HPLC [8] and UV-spectrophotometry [9] have been reported. A UPLC method for the assay of FLU in dosage form in the presence of its related substances was reported [10], but the method did not satisfy the regulatory requirements and did not study the degradation profile, nor characterize the degradation products under stress conditions. Up to date, the literature review did not reveal any study on a stability-indicating HPLC method or/and the identification and characterization of degradation products for FLU in dosage forms. The purpose of this work was to develop a new stability-indicating RP-HPLC method for the estimation of FLU in dosage forms and to characterize the major degradants.

## Experimental

### Materials and Reagents

All reagents were of analytical reagent (AR) grade. HPLC grade methanol and water were procured from Merck, India. Flupirtine maleate (99.66%) was kindly provided by Lupin Pharmaceuticals Limited, Pune, India.

### Instrumentation

An HPLC system (Agilent HPLC Model-1200) equipped with a C_18_ (Agilent BDS, 250 mm × 4.6 mm, 5μ) column, binary pump, rheodyne loop injector with 20 μL, and a photodiode array detector was used. The software used for HPLC data acquisition was EZChrome Elite. A flash chromatograph equipped with silica gel as the column material, and VWD-UV detection (using the software Analogix IF 280 V 5.10) was used for the isolation and purification of degradation products. ^1^H-NMR was recorded on the Varian Unity Inova at 400 MHz (using TMS as internal standard and DMSO-d_6_ as solvent), ^13^C-NMR (Mercury Plus at (abundance 100 MHz), using DMSO-d_6_ as solvent), and mass spectral studies were performed on the API 3000 ABPCIES instrument.

### Forced Degradation Studies

Forced degradation of the FLU drug substance was performed under neutral, acid, alkaline, oxidative, thermal, and photolytic stress conditions. In all stress conditions, the drug concentration used was 1000 μgmL^−1^. After degradation, samples were diluted with mobile phase to a concentration of 50 μgmL^−1^, neutralized to pH 3–4 if necessary, and injected under optimized conditions with the appropriate blank. Blank solutions for each hydrolysis were prepared at the same time with the preparation of stock solutions.

#### Preparation of Stock Solution for Stress Studies

In all of the stress studies, 10 mg of the drug substance was accurately weighed and transferred to a 10 mL volumetric flask, dissolved completely in water, and the solution was diluted up to the mark. The same procedure was used to prepare the stress solutions used for acid hydrolysis, base hydrolysis, and oxidation, respectively, with HCl (0.1–1 N), NaOH (0.01 N), and H_2_O_2_ (0.3%). Initial trials were done with a 0.1 M concentration of HCl and NaOH (0.01 M HCl or NaOH usually preferred if the degradation was drastic in the 0.1 M solutions). For acid degradation, the initial degradation trials were performed in 0.1 M HCl and under these conditions, FLU was stable (< 2%) after three days at room temperature (RT). Hence 1 M HCl was used to induce acid hydrolysis. Thermal degradation was carried out on the solid substance by means of heating the samples over a period in a hot air oven, at 105°C. Photodegradation was carried out on the solid sample according to the procedure described in the following section.

#### Hydrolysis

Stock solutions (1000 μgmL^−1^) were prepared in 0.01 N NaOH (basic), 1 N HCl (acidic), and water (neutral) at room temperature. Samples (500 μL) were withdrawn at different intervals and diluted to 10 mL with mobile phase (50 μgmL^−1^). Samples from the acidic hydrolysis were neutralized with 0.01 M NaOH and samples from the basic hydrolysis were neutralized with 0.01 M HCl.

#### Oxidation

Samples (1 mL) were withdrawn at different times, diluted to 10 mL with mobile phase, and injected under the optimized conditions for analysis. The blank sample was prepared with the same concentration of hydrogen peroxide and analyzed in the same way.

#### Thermal Degradation

To assess the solid-state stability, the sample was heated as a thin layer on a Petri dish at 105°C. Periodically, at 0, 3, 6, 12, 24 hours, 10 mg of the heated sample was weighed, dissolved in water, diluted with mobile phase to 50 μgmL^−1^ and analyzed.

#### Photodegradation

Photodegradation studies were conducted by exposing a solution of the sample to UV radiation at 1.2 million lux-hours for one week (using the photostability chamber Thermolab 400G, New Delhi, India). After degradation, the sample was dissolved in water, diluted with mobile phase to 50 μgmL^−1^ and analyzed under the optimized conditions.

In all of the degradation studies, the percentage degradation of FLU was calculated using the response factor. Peak area was used to calculate the response factor as well as the amount of degradation product formed.

### Synthesis, Purification, and Characterization of Acidic and Basic Degradation Products

#### Acidic Hydrolysis

50 mg of flupirtine maleate was dissolved in 20 mL of water, 5 mL of 50% HCl was added, and the mixture was refluxed for 1 h at 80°C. The reaction mixture was neutralized with alkali to pH 7.0 and kept in a refrigerator overnight. The cloudy precipitate formed was isolated and purified on a silica gel column by using flash chromatography.

#### Basic Hydrolysis

50 mg of flupirtine maleate was dissolved in a mixture of 20 mL of 10% NaOH and refluxed for 2 h at 80°C. The resultant reaction mixture was precipitated by dropwise addition of 5 N HCl. The crude product obtained was recrystallized and purified on a silica gel column by using flash chromatography.

#### Purification

The products were purified by flash chromatography using silica gel as the column material and a mixture of methanol and ethyl acetate (from 5% to 50% methanol) as the mobile phase in gradient mode. VWD-UV detection was carried out for triggering the peak collection and identification of compound fractions. At the end of elution, fractions were combined based upon UV characteristics and TLC, then evaporated in a vacuum evaporator. Three degradation products (D1, D2, and D4) were isolated and characterized by ^1^H-NMR, ^13^C-NMR, and mass spectrometry. These products were spiked with FLU and analyzed under optimized conditions to identify the degradation product in the stress studies. The spectral data of these products are discussed in the following sections.

## Results and Discussion

### Method Development and Optimization of the Chromatographic Conditions

**Fig. 2:**
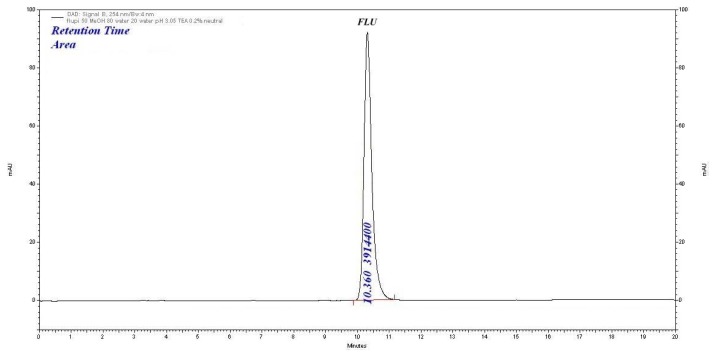
Optimized chromatogram of flupirtine maleate (10.3 min) on a C18 column

In preliminary experiments, the drug was subjected to the reversed-phase mode using a C_18_ column (Agilent, 250 × 4.6 mm, 5μ) and mobile phases consisting of water (pH 3.0 adjusted with orthophosphoric acid) and methanol by varying the % aqueous phase from 10% to 30%. The drug was retained on the column, but the peak shape was not good. It was noted that increasing the % aqueous phase in the mobile phase composition increases the retention time of flupiritine maleate. Based on the suitable retention time for SIAM, the 20% aqueous phase was optimized. To reduce the tailing effect, 0.2% triethylamine (TEA) was added and the pH was adjusted to 3.0 with orthophosphoric acid and the corresponding retention of FLU was 10.3 ± 0.3 min. Finally, the mobile phase of 0.2% *v/v* TEA (pH-adjusted to 3.0 with OPA) and methanol in the ratio of 20:80% *v/v* was optimized. The flow rate was 1.0 mLmin^−1^. The injection volume was 20 μL and the PDA detection wavelength was at 254 nm. The chromatogram obtained in the optimized condition is shown in Fig. 2. It was observed that eight degradation products were formed with retention times 3.9 ± 0.2 min (D1), 4.8 ± 0.2 min (D2), 6.4 ± 0.1 min (D3), 6.8 ± 0.2 min (D4), 8.2 ± 0.2 min(D5), 12.0 ± 0.2 min (D6), 14.1 ± 0.1 min (D7), and 15.0 ± 0.1 min (D8), respectively. The chromatographic resolution among all of the peaks was more than 2. The % degradation was about 5–30% depending on stress conditions.

### Validation Parameters

The method was validated as per ICH (Q2) guidelines with respect to linearity, accuracy, precision, specificity, robustness, limit of detection, and limit of quantification [11].

#### Solution State Stability

The solution state stability of FLU was performed in both water and mobile phases. FLU was estimated every day and carried out for a period of one week. It was concluded that FLU was stable in water for up to 7 days and stable for a period of 3 days in mobile phase.

#### Specificity

Forced degradation studies were performed on FLU to support the specificity of the stability-indicating method. The study was employed on the degradation of FLU by acidic hydrolysis (1 M HCl, 24 h), basic hydrolysis (0.01 M NaOH, 3 h), water hydrolysis (7 days), oxidation (0.3% H_2_O_2_, 24 h), UV light exposure (48 h), and heat (105°C, 24 h). The method was proven to be specific by separating the degradation products formed under the stress conditions.

#### Linearity and Range

The linearity of the detector response to different concentrations of FLU was studied in the range to 20–120 μgmL^−1^ at six different concentrations. Samples were analyzed in triplicate at six different concentrations, such as 20, 40, 60, 80, 100, and 120 μgmL^−1^. The correlation coefficient (*r^2^* value) obtained was 0.9998, indicating a linear response of flupiritine maleate.

#### Accuracy

Accuracy was determined by recovery studies using standard addition. Standard drugs in the range of 80, 100, and 120% of the sample concentration were added to the sample solution. Each concentration was analyzed in triplicate. Results of the recovery studies were between 99.8 to 101.83% and results are shown in Table 1.

#### Precision

Results from the study of intraday and interday precision were obtained by analysis of multiples of the same concentration (50 μgmL^−1^) in the linearity range. The peak area response of each injection was used to calculate the amount of FLU, and the % RSD values for intraday and interday precision were based on the mean and standard deviations. The result was less than 2%, indicating that the method was sufficiently precise; results are shown in Table 2.

#### Robustness

The robustness of the method was determined by the analysis of samples under a variety of changed method conditions, such as flow rate (± 0.1 mL), organic solvent in the mobile phase (± 2%), and pH (± 0.2). The method was robust for all of the conditions for a robust run. The % RSD value for the assay was less than 2% for all of the robust tests. But it was noted that the retention time of FLU was significantly affected (± 1 min) during the change in % organic phase, however the % RSD value was within the limit.

#### Limit of Detection and Limit of Quantification

The LOD and LOQ were determined on the basis of signal-to-noise ratio (S/N). The LOD was taken as the amount for which S/N was 3:1, whereas for the LOQ the S/N was 10:1. The LOD and LOQ were 0.3020 and 0.9153 μgmL^−1^, respectively.

### Forced Degradation

#### Acid-Induced Degradation

**Fig. 3:**
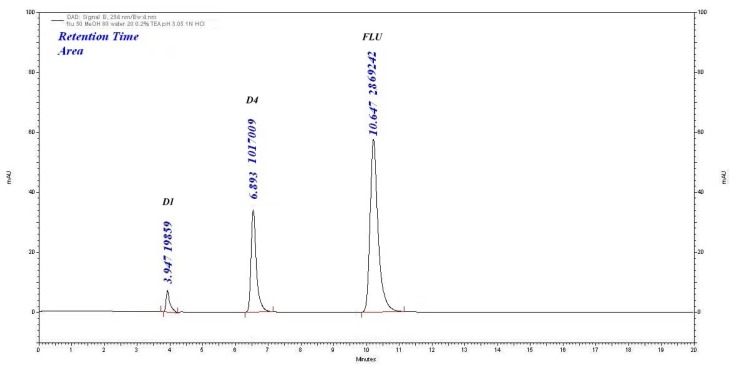
Acid degradation (1 M HCl, 24 h) chromatogram of FLU

Upon treatment with 1 M HCl at RT for 24 h, two degradation peaks (D1, D4) were observed. The percentage degradation was 28.43% in 24 h. The assay value of FLU was 71.45%, as shown in Fig. 3.

#### Base-Induced Degradation

**Fig. 4:**
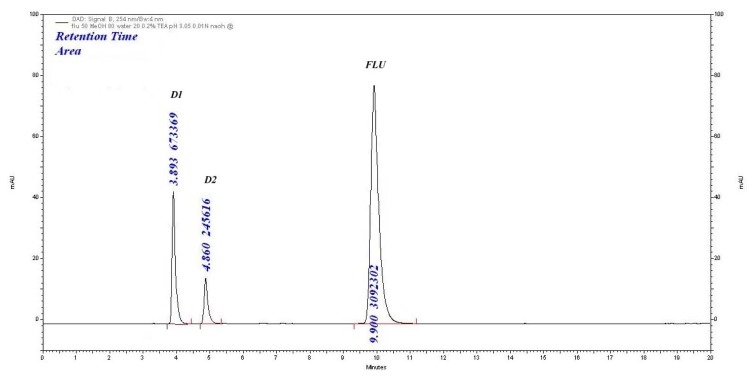
Base degradation (0.01 M NaOH, 3 h) chromatogram of FLU

In 0.01 M NaOH as the stress condition, two degradation products (D1 and D2) at 3.89 min and 4.86 min were observed with the percentage degradation of 6.16% and 22.80%, respectively. The assay of FLU was found to be 70.13% shown in Fig. 4.

#### Oxidative Degradation

**Fig. 5:**
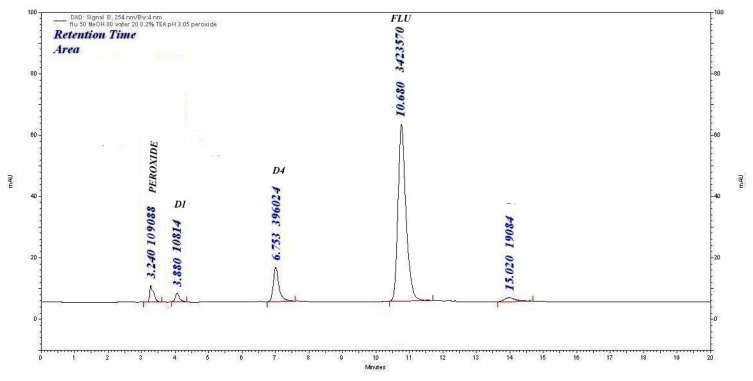
Peroxide degradation (0.3% H_2_O_2_, 24 h) chromatogram of FLU

Upon treatment of FLU with 0.3% H_2_O_2_ at RT for 24 h, the degradation was 22.11% with the formation of three products (D1, D4, and D8). The assay of FLU was 76.90%, as shown in Fig. 5.

#### Thermal Degradation

**Fig. 6:**
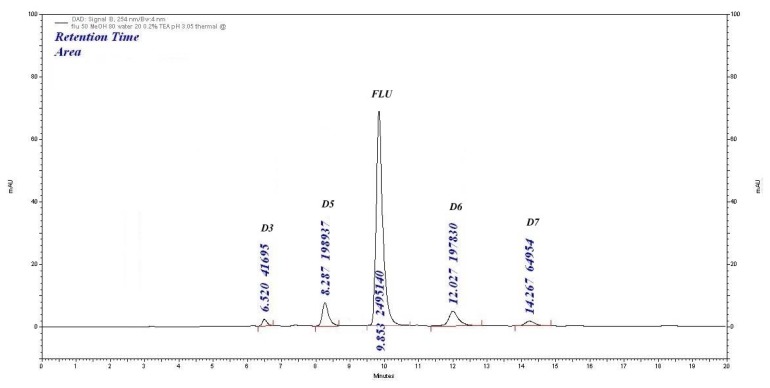
Thermal degradation (105°C, 24 h) chromatogram of FLU

When the drug was exposed to dry heat in an oven at 105°C for 24 h, four degradation products were formed at 6.52 min (D3), 8.28 min (D5), 12.02 min (D6), and 14.26 min (D7) with significant changes in the peak area of FLU. The degradation was 20.17%. The % assay value was 72.66%. The chromatogram is shown in Fig. 6.

#### Neutral Degradation

The drug did not show any degradation even after 7 days at room temperature and the assay of the active substance was found to be 98.98%.

#### Photolytic Degradation

**Fig. 7:**
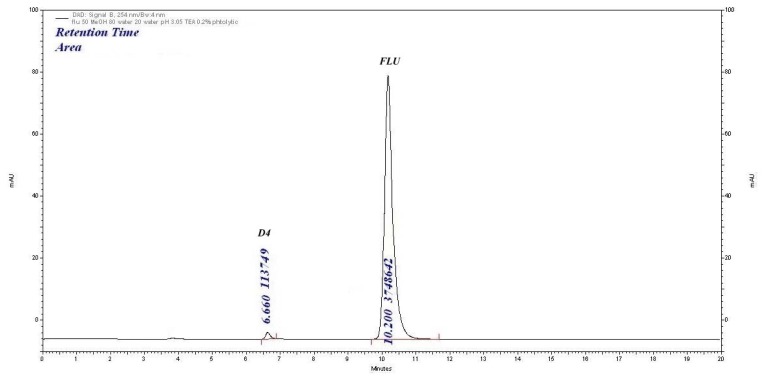
Photolytic degradation (48 h, UV-light) chromatogram of FLU

The drug was exposed to photolytic degradation under UV light for 48 h, one degradation product (D4) was at a retention time of 6.6 min and the percentage degradation was 9.06% as shown in Fig. 7. The assay of FLU in the photo-degraded sample was found to be 86.6%.

The detailed percentage degradations of each degradation product in all stress conditions are shown in Table 3. The mass balance was between 98–99% for both acidic and basic hydrolysis. All degradation products were adequately separated, thus the method was more selective and specific. The peak purity was more than 0.999. The study revealed that FLU was more sensitive to all stress conditions. No degradation product was observed after 15 min. FLU was more labile to degradation in the presence of base, peroxide, acid, and UV light.

### Identification of Degradants

In these stress studies, a total of eight degradation products (D1–D8) were observed for flupiritine maleate, among these products three degradation products, D1 (acidic, basic, and peroxide), D2 (basic), and D4 (acidic, peroxide, and photolytic) were synthesized and purified by flash chromatography (Table 3). The obtained D1, D2, and D4 products were characterized by ^1^H-NMR, ^13^C-NMR, and mass spectral data. Spike analysis of degradants with standard flupirtine maleate solution confirmed the retention times of the elucidated degradants. The retention for D1, D2, and D4 were identified as 3.9, 4.8, and 6.8 ±0.2 min, respectively. D1 was observed in the acidic and basic hydrolysis and peroxide stress studies. It was characterized as {2-amino-6-[(4-fluorobenzyl)amino]pyridin-3-yl}carbamic acid. The D2 degradant belonged to the basic stress condition alone and was chemically identified as *N*^6^-(4-fluorobenzyl)pyridine-2,3,6-triamine. The D4 degradant was observed under acidic, peroxide, and photolytic stress conditions and characterized as 5-[(4-fluorobenzyl)amino]-1,3-dihydro-2*H*-imidazo[4,5-*b*]pyridin-2-one.

#### Degradant 1 (D1) {2-amino-6-[(4-fluorobenzyl)amino]pyridin-3-yl}carbamic acid

Formula: C_13_H_13_FN_4_O_2_, molecular weight: 276.1, ^1^H-NMR (CDCl_3,_ δ ppm): 3.81 (coupled, 2H, CH_2_), 5.61 (s, 2H, NH_2_), 6.91–7.18 (m, 4H, fluorophenyl), 7.52–7.79 (m, 2H, pyridine), 12.12 (s, 1H, CO-NH), 16.2 (s, 1H, COOH). ^13^C-NMR (CDCl_3_, δ ppm): 167.5 (C=O), 158.2, 141.6, 140.9, 138.11, 136.7, 131.2, 131.2, 131.0, 130.5, 128.9, 127.7, 126.4, 66.4. Mass (ESI, positive mode): 277 (M+H).

#### Degradant 2 (D2) N^6^-(4-fluorobenzyl)pyridine-2,3,6-triamine

Formula: C_12_H_13_FN_4_, molecular weight: 232.25, ^1^H-NMR (CDCl_3,_ δ ppm): 3.65 (s, 2H, CH_2_), 5.54, 5.65 (s coupled, 4H, NH_2_), 7.02–7.23 (m, 4H, fluorophenyl), 7.59–7.81 (m, 2H, pyridine). ^13^C-NMR (CDCl_3_, δ ppm): 158.9, 142.1, 140.7, 138.4, 137.2, 131.5, 131.4, 131.2, 130.1, 129.4, 127.6, 126.4, 64.9. Mass (ESI, positive mode): 233 (M+H).

#### Degradant 3 (D4) 5-[(4-fluorobenzyl)amino]-1,3-dihydro-2H-imidazo[4,5-b]pyridin-2-one

Formula: C_13_H_11_FN_4_O, molecular weight: 258.1, ^1^H-NMR (CDCl_3,_ δ ppm): 3.52 (cpx coupled, 2H, CH_2_), 6.95–7.23 (m, 4H, fluorophenyl), 7.66–7.92 (m, 2H, pyridine), 8.43, 9.42 (broad, 2H, NH exchangable). ^13^C-NMR (CDCl_3_, δ ppm): 171.4 (C=O), 155.3, 141.1, 140.5, 138.3, 137.9, 132.5, 131.5, 131.1, 129.8, 128.6, 127.1, 126.8, 64.4. Mass (ESI, positive mode): 259 (M+H).

## Conclusion

An isocratic, specific, stability-indicating RP-HPLC method has been developed for the estimation of flupirtine maleate in pharmaceutical dosage forms and is validated as per ICH (Q2) guidelines. The method is specific and unaffected by the presence of seven degradants, indicating the method’s suitability as a stability-indicating assay of flupirtine maleate, which is suitable to identify and quantify all of the degradation products.

## Figures and Tables

**Tab. 1:** Accuracy of the method (n = 6)

Conc. (μgmL^−1^)	Recovery level	Amount Added (μgmL^−1^)	Amount Recovered (μgmL^−1^)	% Recovery	% RSD
	80%	40	39.57 ± 0.09	98.92	0.23
50	100%	50	50.49 ± 0.10	100.98	0.20
	120%	60	59.31 ± 0.21	98.85	0.36

**Tab. 2:** Intraday and Interday precision of the method

Conc. (μgmL^−1^)	Intraday (mean ± SD; n = 6)	Intraday (% RSD)	Interday (mean ± SD; n = 6)	Interday (% RSD)
25	24.24 ± 0.26	1.07	24.78 ± 0.32	1.28
50	50.16 ± 0.45	0.89	49.96 ± 0.57	1.16
100	99.86 ± 0.74	0.74	100.06 ± 0.98	0.98

**Tab. 3:** Degradation data of flupirtine maleate under stress studies (FLU)

Stress Condition	No. of Degradants	% Degradation (Dx) (x = 1–8)^a^								% Assay of FLU

		*D1*	*D2*	*D3*	*D4*	*D5*	*D6*	D7	D8	
1 M HCL (24 h)	2	3.41	–	–	25.02	–	–	–	–	71.45
0.01 M NaOH (3 h)	2	6.16	22.80	–	–	–	–	–	–	70.13
0.3% H_2_O_2_ (24 h)	2	2.71	–	–	17.09	–	–	–	2.31	76.90
Heat 105°C (24 h)	4	–	–	3.04	–	7.96	5.63	3.54	–	72.66
UV-light (48 h)	1	–	–	–	9.06	–	–	–	–	86.60
Water (7 Days)	0	–	–	–	–	–	–	–	–	98.98

a Degradation products R_t_: 3.9 ± 0.2min (D1), 4.8 ± 0.2 min (D2), 6.4 ± 0.1 min (D3), 6.8 ± 0.2min (D4), 8.2 ± 0.2min(D5), 12.0 ± 0.2 min (D6),14.1 ± 0.1 min (D7), 15.0 ± 0.1 min (D8).
